# Wearable sensors in patient acuity assessment in critical care

**DOI:** 10.3389/fneur.2024.1386728

**Published:** 2024-05-09

**Authors:** Jessica Sena, Mohammad Tahsin Mostafiz, Jiaqing Zhang, Andrea E. Davidson, Sabyasachi Bandyopadhyay, Subhash Nerella, Yuanfang Ren, Tezcan Ozrazgat-Baslanti, Benjamin Shickel, Tyler Loftus, William Robson Schwartz, Azra Bihorac, Parisa Rashidi

**Affiliations:** ^1^Department of Computer Science, Federal University of Minas Gerais, Belo Horizonte, Brazil; ^2^Department of Electrical and Computer Engineering, University of Florida, Gainesville, FL, United States; ^3^Division of Nephrology, Hypertension, and Renal Transplantation, Department of Medicine, College of Medicine, University of Florida, Gainesville, FL, United States; ^4^Intelligent Clinical Care Center, University of Florida, Gainesville, FL, United States; ^5^Department of Medicine, Stanford University, Stanford, CA, United States; ^6^J. Crayton Pruitt Family Department of Biomedical Engineering, University of Florida, Gainesville, FL, United States; ^7^Department of Surgery, College of Medicine, University of Florida, Gainesville, FL, United States

**Keywords:** intensive care unit, ICU, accelerometer, acuity assessment, electronic health record, deep learning, artificial intelligence

## Abstract

Acuity assessments are vital for timely interventions and fair resource allocation in critical care settings. Conventional acuity scoring systems heavily depend on subjective patient assessments, leaving room for implicit bias and errors. These assessments are often manual, time-consuming, intermittent, and challenging to interpret accurately, especially for healthcare providers. This risk of bias and error is likely most pronounced in time-constrained and high-stakes environments, such as critical care settings. Furthermore, such scores do not incorporate other information, such as patients’ mobility level, which can indicate recovery or deterioration in the intensive care unit (ICU), especially at a granular level. We hypothesized that wearable sensor data could assist in assessing patient acuity granularly, especially in conjunction with clinical data from electronic health records (EHR). In this prospective study, we evaluated the impact of integrating mobility data collected from wrist-worn accelerometers with clinical data obtained from EHR for estimating acuity. Accelerometry data were collected from 87 patients wearing accelerometers on their wrists in an academic hospital setting. The data was evaluated using five deep neural network models: VGG, ResNet, MobileNet, SqueezeNet, and a custom Transformer network. These models outperformed a rule-based clinical score (Sequential Organ Failure Assessment, SOFA) used as a baseline when predicting acuity state (for ground truth we labeled as unstable patients if they needed life-supporting therapies, and as stable otherwise), particularly regarding the precision, sensitivity, and F1 score. The results demonstrate that integrating accelerometer data with demographics and clinical variables improves predictive performance compared to traditional scoring systems in healthcare. Deep learning models consistently outperformed the SOFA score baseline across various scenarios, showing notable enhancements in metrics such as the area under the receiver operating characteristic (ROC) Curve (AUC), precision, sensitivity, specificity, and F1 score. The most comprehensive scenario, leveraging accelerometer, demographics, and clinical data, achieved the highest AUC of 0.73, compared to 0.53 when using SOFA score as the baseline, with significant improvements in precision (0.80 vs. 0.23), specificity (0.79 vs. 0.73), and F1 score (0.77 vs. 0.66). This study demonstrates a novel approach beyond the simplistic differentiation between stable and unstable conditions. By incorporating mobility and comprehensive patient information, we distinguish between these states in critically ill patients and capture essential nuances in physiology and functional status. Unlike rudimentary definitions, such as equating low blood pressure with instability, our methodology delves deeper, offering a more holistic understanding and potentially valuable insights for acuity assessment.

## 1 Introduction

Acuity refers to the severity of a patient’s condition, concomitant with the priority assigned to patient care in a critical care setting. Patients in the intensive care unit (ICU) exhibit volatile physiological patterns and the potential for developing life-threatening conditions in a short period. Therefore, the timely recognition of evolving illness severity is of immense value in the ICU. Swift and precise assessments of illness severity can identify patients requiring the administration of immediate life-saving interventions (1). Furthermore, these assessments can guide collaborative decision-making involving patients, healthcare providers, and families in determining care goals and optimizing resource allocation (2). Patient acuity is a foundational concept in critical care that ensures patient needs are met with precision, safety, and efficiency. Accurate acuity assessments are crucial for guiding clinical interventions, optimizing staffing ratios, and ensuring the presence of adequately trained personnel to address the needs of high-acuity patients (3, 4). From management and fiscal perspectives, an accurate understanding of in-patient acuity levels permits effective budgeting and resource allocation (5).

Traditional, manual, threshold-based scoring systems such as the Acute Physiology and Chronic Health Evaluation (APACHE) (6), the Simplified Acute Physiology Score (SAPS) (7), Sequential Organ Failure Assessment (SOFA) (8), Modified Early Warning Score (MEWS) (9) and others, have been developed to predict the risk of mortality in ICU patients and, by extension, gauge the complexity of their care needs (6). These tools evaluate physiological parameters, laboratory results, and other pertinent clinical information. However, static variable thresholds and additive scores have lesser predictive accuracy for outcomes of interest, and they tend to use a few rudimentary biomarkers to represent complex disease states.

Recent studies in clinical informatics have highlighted the efficacy of automated machine learning methods in leveraging comprehensive data from electronic health record (EHR) systems. EHR encompasses a variety of patient-level data categories, including demographic information, diagnoses, procedures, vital signs, medications, and laboratory measurements. The studies have emphasized the potential of machine learning in transforming healthcare by enhancing clinical decision-making processes and patient care. For example, Clifton et al. (10) have discussed the use of health informatics systems based on machine learning in clinical patient management, demonstrating the relevance of these technologies in healthcare settings. Additionally, Wang et al. (11) have supported this idea by showcasing the widespread adoption of machine learning in mining EHRs to advance clinical research and practice.

Furthermore, Hu et al. (12) and Miotto et al. (13) have investigated the application of automated machine learning in distinguishing between types of cancers and predicting patient outcomes based on EHR data. These studies have underscored the potential of machine learning to accelerate workflow, enhance performance, and improve the accessibility of artificial intelligence in clinical research. Moreover, the work by Wang et al. (14) has highlighted the opportunity presented by EHR data for patient similarity assessment and personalized medicine through machine learning. Advanced algorithms using deep learning techniques have proven superior to conventional bedside severity evaluations in predicting in-hospital deaths, an indirect measure of immediate patient acuity. However, these systems are limited to physiological data captured within the EHR and neglect other significant aspects impacting the patient, such as mobility and functional status (1).

To overcome these limitations, Davoudi et al. (15) explored the benefits of augmenting traditional ICU EHR-based data with continuous and pervasive sensing technology. The study gathered detailed information on ICU patients’ activity levels, environmental factors, and behaviors by combining data from wearable sensors, light and sound sensors, and a camera. This multi-sensor approach provided a holistic perspective on patient care and monitoring, facilitating thorough analysis of delirium classification in critical conditions. Wearable device data significantly contributed to the study’s results by offering valuable insights into patients’ activity levels, movement patterns, and functional status. The study shows that integrating wearable sensor data with other modalities enables a comprehensive assessment of patients’ behaviors and conditions in the ICU, potentially leading to advancements in patient care and monitoring. Inspired by the positive impact of these novel clinical data streams, Shickel et al. (1) proposed to augment EHR data with continuous activity measurements via wrist-worn accelerometer sensors to predict hospital discharge status as a proxy for acuity. The study employs deep learning techniques, specifically single-layer recurrent neural networks (RNN) with gated recurrent units (GRU), to process sequential data and make predictions about patient illness severity. The findings suggest that integrating pervasive sensing data with conventional EHR data can enhance real-time acuity estimation for critically ill patients. Furthermore, they propose that additional investigation and integration of even more innovative data streams could offer further benefits in this regard. Our previous work also highlighted the efficiency of accelerometer data in predicting pain levels (16).

**Figure 1:**
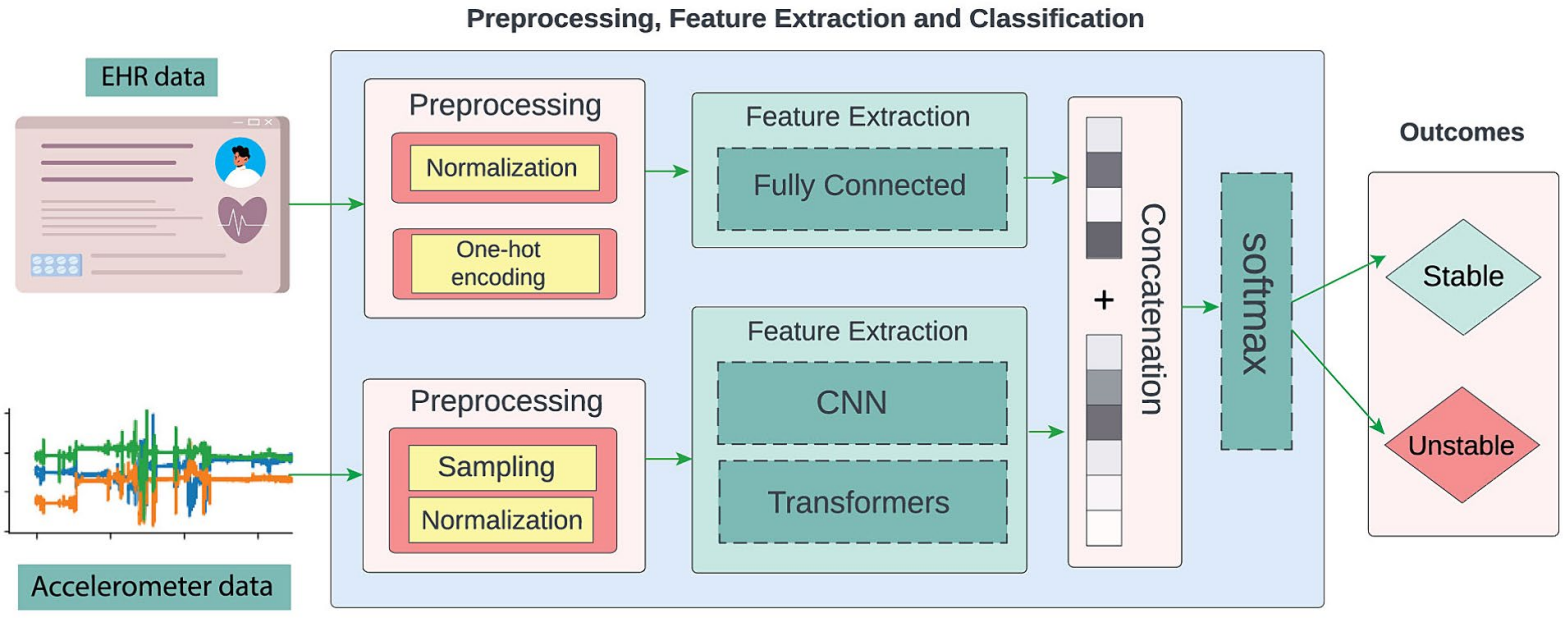
The proposed approach is an end-to-end neural network system that leverages accelerometer and EHR data to assess patient acuity, discerning between stable and unstable states.

In this work, we differ from the current literature by proposing to evaluate the viability of using accelerometer and EHR data to assess patients’ acuity directly instead of using patient discharge status as a proxy. Following the same acuity phenotyping approach proposed by Ren et al. (17), the goal is to discern the patient’s state as stable or unstable. To achieve this, we have developed an end-to-end deep learning pipeline based on accelerometer and EHR data (Figure 1).

We evaluated five different neural network architectures, namely, VGG (18), ResNet (19), MobileNet (20), SqueezeNet (SENet) (21), and a custom Transformer-based network (22) since both convolutional neural networks (CNNs) and Transformers architectures are well-accepted in the sensor-based human activity recognition field (23–27). The CNN architecture can detect patterns regardless of their position in the sequence and extract both simple and complex movement patterns due to its hierarchical structure. On the other hand, Transformers are advantageous for processing accelerometer data because their self-attention mechanism can capture long-term dependencies and weigh the importance of different elements in a temporal sequence (28). Consequently, each patient’s movement can be contextualized in relation to the other movements within a time window, directing the network’s attention to the key movement patterns for assessing the patient’s condition.

## 2 Materials and methods

### 2.1 Study cohort

The data used in this research were sourced from adult patients admitted to one of nine specialized ICUs at the University of Florida (UF) Health Shands Hospital main campus in Gainesville, Florida, in compliance with all relevant federal, state, and university laws and regulations. Approval for the study was granted by the University of Florida Institutional Review Board under IRB201900354 and IRB202101013. Before enrolling patients in the study, written informed consent was obtained from all participants. In cases where patients could not provide informed consent, consent was obtained from a legally authorized representative (LAR) acting on their behalf. Eligible participants were individuals aged 18 and older who were admitted to an ICU and expected to remain there for at least 24 h. Patients were enrolled independent of their disease, and their diagnoses were unknown to the study team at the time of recruitment, which took place in person by trained clinical research coordinators. Those who could not provide LAR or self-consent, were expected to be transferred or discharged from the ICU within 24 h, were receiving comfort measures only, were unable to provide informed consent at baseline, and those necessitating contact or isolation precautions, were excluded. Also excluded from this study were patients who expired within 24 h of recruitment or from whom we could not collect accelerometer data due to the presence of intravenous lines, wounds, other hospital equipment, or the patient’s choice to opt out of accelerometer placement. Accelerometers were still applied to intubated and sedated patients.

Datasets were acquired from 87 critically ill patients between June 2021 and February 2023. Figure 1 depicts the data sources: EHR and accelerometer readings. Patients wore Shimmer3 (28) or ActiGraph wGT3X-BT (29) accelerometers on one of their wrists. The accelerometers used in this study capture direction and magnitude of acceleration along 3 axes. The accelerometers convey information on the patient’s arm’s direction and intensity of movement as well as rotational position through continuous measurement of linear acceleration and angular velocity of the device. These types of devices capture various aspects of movement and activity, offering insights into physical dynamics such as speed, direction, and intensity of motion. These measurements enable the quantification of movement patterns and activity levels with a high degree of precision and detail. In this work we did not include clinical information reflected at the motor level such as assessments of muscle strength, coordination, balance, and overall mobility. Accelerometer readings were taken for a maximum of 7 days or until the patient’s discharge from the ICU, whichever came first. During this time, the study team performed daily visits to ensure that the device was correctly positioned on the patient’s wrist and requested that the nursing staff document any times when the device was removed. All known removal and reapplication times were documented as device downtimes to be excluded from analysis. Conservative estimations were used if the exact removal time was unknown. We gathered 9,286 h of accelerometer data, with an average of approximately 107 h per patient. Data from ActiGraph devices were retrieved using the ActiLife toolbox.^1^ Data from the Shimmer device was uploaded and exported to a secure server via the Consensys software.^2^

Using a daily pipeline, UF’s Integrated Data Repository service extracted clinical data relevant to the patient’s acuity state from the EHR. This information included demographics such as age, sex, race, height, weight, length of stay, medications, and physiological signals like blood pressure, heart rate, oxygen saturation (SpO2), respiratory device, continuous renal replacement therapy, blood transfusion, pain score, Braden score (30), and acute brain dysfunction status (whether the patient was in a coma, experiencing delirium, or had normal cognitive status) (31).

### 2.2 Data processing

In this work, we employed supervised machine learning algorithms. This family of algorithms learns the relationship between data and a target. In our case, the target is a patient’s acuity state in a certain time range. In order to train these algorithms, each sample (time window) needs to be labeled with the correct acuity state so the algorithm can learn the patterns and correlations between data and the target. To phenotype the patient acuity state as stable or unstable, we applied the method devised by Ren et al. (17), which determines transitions in acuity status within the ICU. To capture the relevant data (accelerometer and clinical data) leading up to each assessment, we established a consecutive and non-overlapping 4-h segmentation window that concluded immediately before the acuity evaluation, to reflect patients’ status. For every 4 h leading up to the assessment, patients—excluding those who had passed away or were already discharged alive—were identified as unstable or stable. A patient was labeled as unstable if they required any of the following life-supportive therapies: vasopressors (epinephrine, vasopressin, phenylephrine, norepinephrine, droxidopa, or ephedrine), mechanical ventilation, continuous renal replacement therapy, or a massive blood transfusion (defined as at least ten units in the previous 24 h), as previously described. If none of these conditions were met, the patient was considered stable.

To address the varying sampling frequencies of the accelerometer data, we downsampled all accelerometer segmented windows to a consistent 10 Hz sampling frequency. This downsampling not only ensures uniformity in the input data rate, facilitating more accurate analysis but also limits the maximum length of the accelerometer sequence to 144,000 (14,400 s x 10 Hz) to avoid extremely long sequences. Additionally, accelerometer values were normalized to a range of [0, 1] in a sample-wise fashion (min and max values were calculated per sample) to accommodate the requirements of the deep learning methods evaluated in our study. Similarly, numerical demographic data, such as age, was normalized to the [0, 1] range, while categorical demographic information like sex and race was one-hot encoded. All clinical data consisted of time series captured within 4-h windows, each varying in length.

### 2.3 Deep learning models

The prediction models evaluated in this work were VGG (18), ResNet (19), MobileNet (20), SqueezeNet (SENet) (21), and a custom Transformer-based network (22). The selection was grounded in their capabilities: VGG and ResNet for their depth, MobileNet, and SENet for their small number of parameters compared to ResNet and VGG, thus making them a suitable choice for edge deployment and for reducing the decision-making latency, which is crucial if deployed in the ICU setting. The transformer was selected for its unique attention mechanism, which enables modeling long-range dependencies in input signals. VGG, ResNet, MobileNet, and SENet were initially designed for image classification and required an architecture adaptation to suit accelerometer data. We tailored the original models to process 1D time series while preserving the fundamental layer-wise structure and defining characteristics. It entailed replacing 2D convolution, average pooling, and max pooling layers with their 1D counterparts and adjusting input channel configurations to match our data dimensions. For ResNet, SqueezeNet, and MobileNet, we retained essential components such as residual blocks (in ResNet), Squeeze-and-Excitation blocks (in SqueezeNet), and Depthwise Separable Convolution blocks (in MobileNet), with modifications primarily consisting of substituting 2D convolution and pooling filters with their 1D counterparts and updating channel parameters. The fully connected layers were kept unchanged. To further aggregate clinical and demographic features into the classification pipeline, we concatenated them with the dense features extracted from the fully connected layer.

**Figure 2:**
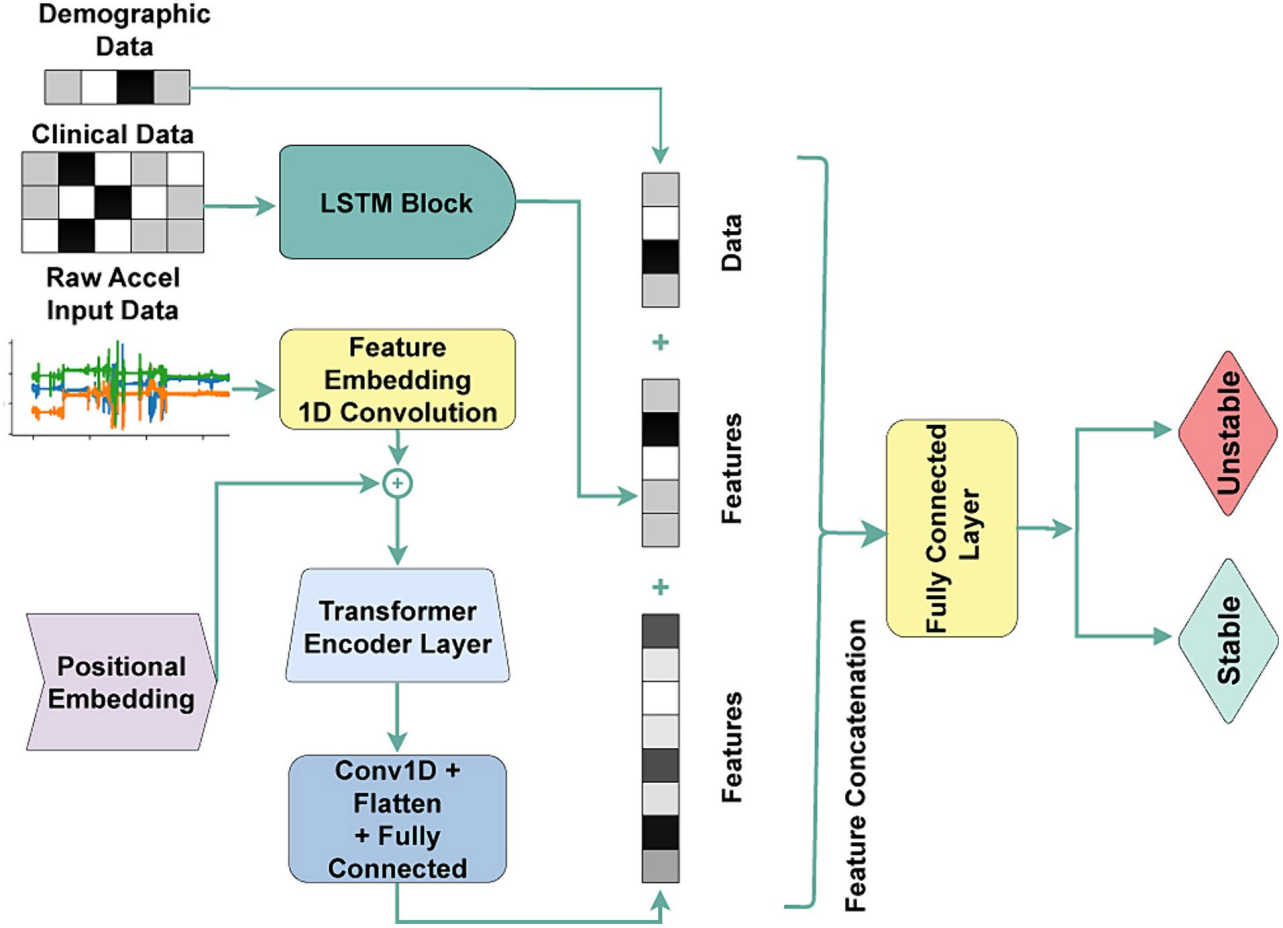
The transformer architecture is used for the acuity state classification task. Accel: Accelerometer Sensor, Conv1D: 1D Convolution, Flatten: 2D-to-1D flattening layer, Fully Connected: fully connected layer.

In contrast, Transformer architectures are innately suited for sequence data processing due to their self-attention mechanism and parallel processing capabilities. In our methodology, we extracted sequential feature embeddings from raw accelerometer sensor data using a feature embedding convolution layer with a kernel size of 5 and 64 channels. We then provided the extracted features, followed by adding positional encodings to capture the temporal order of the data into a Transformer encoding layer. We further processed the extracted contextual features through another set of convolution and fully connected layers to enable our downstream classification tasks. We concatenated clinical and demographic features with the dense features extracted from the fully connected layer, like the approach adopted in earlier models. The model architecture is demonstrated in Figure 2.

### 2.4 Experiments

In assessing the deep learning models, we implemented a thorough evaluation protocol aimed at ensuring reliability and transparency, with a particular emphasis on subject independence. This protocol combined two established methods: 5-fold cross-validation and the holdout approach.

Initially, the holdout method divided the dataset into a development set (70%) and a separate holdout test set (30%), adhering to subject independence principles. The 5-fold cross-validation was then applied within the development set to facilitate robust hyperparameter optimization and guard against potential overfitting. This step was crucial for obtaining a reliable performance estimate, especially given our dataset’s limited size. Within each fold, distinct training and validation datasets were created, ensuring that each patient’s data were exclusively assigned to either the training or validation set used in that fold. This approach maintained the integrity of the evaluation process and upheld the principle of subject independence throughout.

The models underwent training and validation using the development dataset to determine the most effective hyperparameters. Following the completion of this step, they were assessed using the holdout test set to gauge their ability to generalize to unseen data.

**Figure 3:**
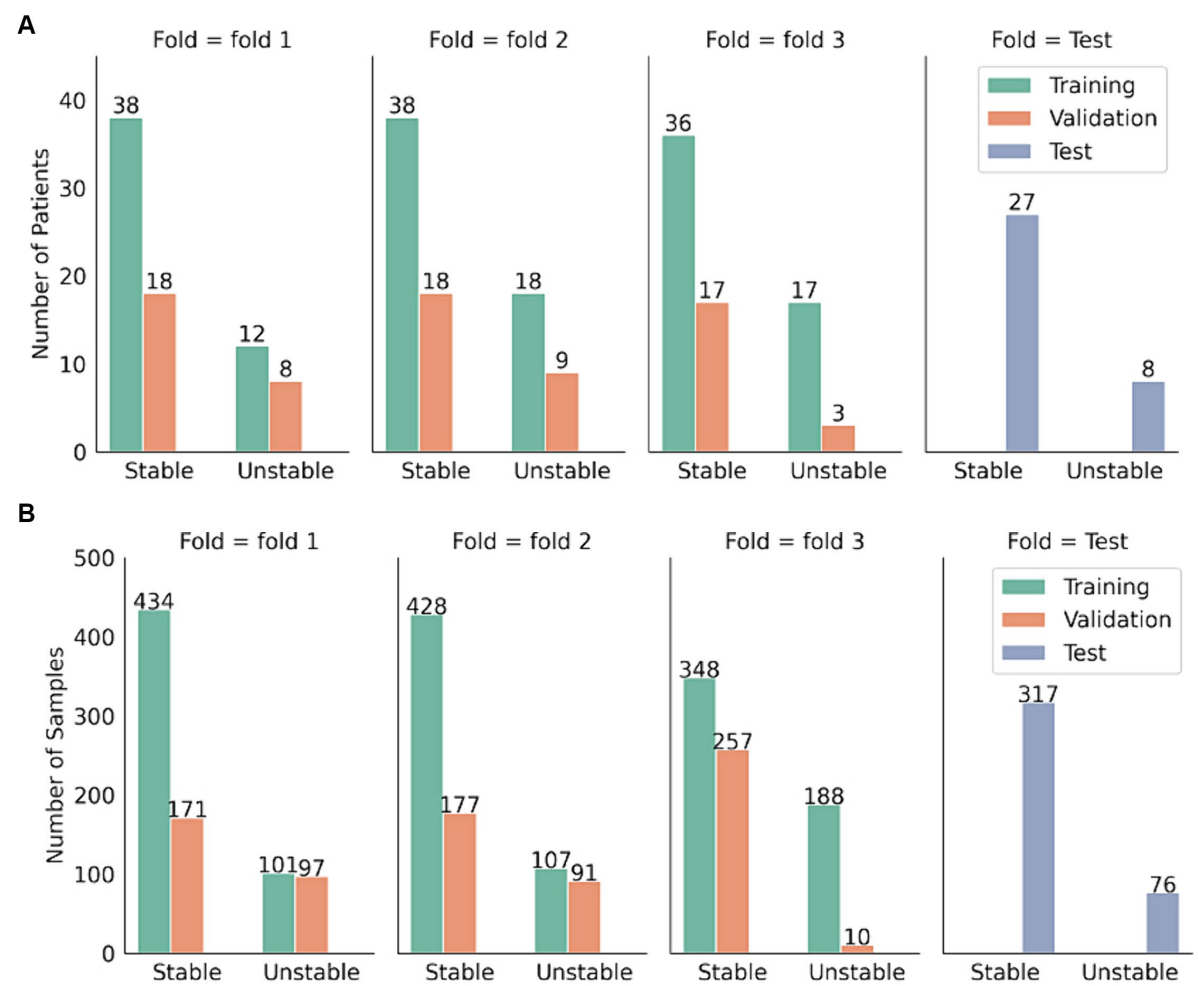
Distribution of patients and samples distribution in the test set and the three-folded development set. **(A)** Sample distribution. **(B)** Patient distribution.

Figure 3 shows the patient and sample distribution for our dataset.

During the 5-fold cross-validation process, we utilized Optuna (32) to search over the hyperparameters rather than a traditional grid search. Optuna reduces the runtime by pruning fewer promising trials during runtime. For every set of hyperparameters, we maximized the area under the ROC curve (AUC) for each fold. After deriving the AUC for every fold, we calculated the mean and standard deviation of these values over all folds of the 5-fold cross-validation. The hyperparameters yielding the highest mean validation AUC across all folds were deemed optimal and were used to train the final model.

In addition to using deep neural networks, we also incorporated the SOFA score as a rule-based scoring system into our evaluation process as a baseline. The SOFA score, well-established in assessing patients in ICUs, provides an objective and standardized means of tracking a patient’s condition over time. These properties make the SOFA score an indicator of the acuity state assessment task. To measure the acuity states, we scaled the SOFA scores within the range of [0, 1] using min-max normalization. We treated these normalized scores as probability values and utilized the Youden index (33) to determine the optimal threshold for classifying the normalized scores and generating predictions.

Once the models were trained using optimized hyperparameters over the entire training cohort, we assessed its performance on a holdout test set using bootstrapping with replacement. We created 100 synthetic bootstrapped versions of the holdout test set samples. These bootstrapped test sets were of the same length as the original test set. The model’s performance was then calculated on all bootstraps. We reported the median and 95% confidence interval (CI) of several performance metrics: AUC, precision, sensitivity, specificity, and F1 score. The *p*-value was calculated to assess the statistical significance of the observed performance metrics values against a null hypothesis that was no better than the previous setups (34).

Finally, we performed SHAP (SHapley Additive exPlanations) (35) analysis on the best-performing models to interpret relative feature importance, providing insights into how various features contribute to model predictions. This analysis aids in understanding the model’s decision-making process and can guide further refinement or feature engineering efforts.

## 3 Results

### 3.1 Participants

We involved 87 patients based on our inclusion and exclusion criteria. The demographic and clinical variables of the patients analyzed were detailed in Table 1, while Table 2 provided a breakdown of demographics categorized by stable and unstable conditions. The distribution of patients by race and gender are approximately the same in both development and test sets. The average age was slightly higher in the development cohort, though not significantly so. Heights were similar between cohorts, but the mean weight was notably greater in the development cohort. Length of stay did not significantly differ between cohorts. Notable differences in disease prevalence included a higher occurrence of cancer and diabetes in the test cohort, while liver-related diseases were more frequent in the development cohort.

**Table 1:** Patients characteristics.

Variables	Development Cohort (*N* = 60)	Test cohort (*N* = 27)	*p*-value
Female sex, *N* (%)	22 (36.7%)	9 (33.3%)	0.76
Hispanic ethnicity, *N* (%)	8 (13.3%)	2 (7.4%)	0.42
Age in years, mean (SD)	58.4 (15.9)	52.2 (18.3)	0.12
Height in cm, mean (SD)	173.6 (9.1)	172.4 (8.5)	0.56
Weight in kgs, mean (SD)	87.2 (23.6)	77.8 (15.0)	0.06
Length of stay in days, median (25th, 75th percentile)	11.0 (6.0, 29.0)	13.0 (8.0, 23.0)	0.60
Race: *N* (%)			
White	49 (81.7%)	18 (66.7%)	0.12
African American	9 (15.0%)	3 (11.0%)	0.63
Other	2 (3.3%)	6 (22.2%)	<0.05
Comorbidities: *N* (%)			
Cancer	0 (0.0%)	6 (22.2%)	<0.05
Cerebrovascular disease	8 (13.3%)	4 (14.8%)	0.85
Dementia	1 (1.7%)	2 (7.4%)	0.18
Paraplegia hemiplegia	6 (10.0%)	2 (7.4%)	0.70
Congestive heart failure	7 (11.7%)	2 (7.4%)	0.55
Chronic obstructive pulmonary disease	4 (6.7%)	3 (11.1%)	0.48
Diabetes	7 (11.7%)	6 (22.2%)	0.20
Liver disease	15 (25.0%)	5 (18.5%)	0.51
Peptic ulcer	2 (3.3%)	0 (0.0%)	0.34
Renal disease	9 (15.0%)	4 (14.8%)	0.98

SD, standard deviation; *N*, number. In our analysis, we employed two distinct statistical tests to examine the differences between the development cohort and the test cohort. For the continuous variables, we used Welch’s *t*-test, while for the categorical variables, we used the two-proportion *z*-test, appropriately.

**Table 2:** Distribution of demographic variables of encounters (recorded every four hours) stratified by class labels (stable, unstable).

Variables	Stable encounters (*N* = 434)	Unstable encounters (*N* = 101)	*p*-value
Female sex, *N* (%)	187 (43.0%)	16 (15.8%)	<0.05
Hispanic ethnicity, *N* (%)	64 (14.8%)	4 (4.0%)	<0.05
Age in years, mean (SD)	59.2 (16.7)	57.5 (13.4)	0.34
Height in cm, mean (SD)	171.2 (9.1)	178.6 (7.2)	<0.05
Weight in kg, mean (SD)	83.6 (21.50)	97.5 (20.2)	<0.05
Length of stay in days, median (25th, 75th percentile)	16.0 (8.0, 31.0)	29.0 (12.0, 33.0)	<0.05
Race, *N* (%)			
White, *N* (%)	336 (77.4%)	85 (84.2%)	0.97
African American, *N* (%)	42 (9.7%)	16 (15.8%)	0.07
Other, *N* (%)	56 (100.0%)	0 (0.0%)	<0.05

SD, standard deviation; *N*, number.

### 3.2 Experiment results

We evaluated the performance of five deep learning models on different combinations of feature sets: accelerometer data only (Accel), accelerometer data with demographics (Accel + Demo), accelerometer data with clinical information (Accel + Clinical), and a combination of accelerometer data, demographics, and clinical information (Accel + Demo + Clinical). We refer to demographics as the features of age, sex, race, height, and weight and as clinical data, the length of stay, blood pressure, heart rate, SpO2, pain score, Braden score, and cognitive status. In addition, we used the SOFA score as a baseline to compare performances across the rule-based and deep learning-based methods. We also evaluate the combination of demographics and clinical data (Demo + Clinical) to evaluate the accelerometer’s contribution to the acuity status assessment. The results are summarized in Table 3.

**Table 3:** The best results reported as average and 95% confidence interval in each scenario.

	Model	AUC (95% CI, *p*-value)	Precision (95% CI, *p*-value)	Sensitivity (95% CI, *p*-value)	Specificity (95% CI, *p*-value)	F1-score (95% CI, *p*-value)
SOFA score	–	0.53 (0.48–0.58)	0.23 (0.19–0.28)	0.30 (0.22–0.38)	0.76 (0.69–0.82)	0.66 (0.61–0.72)
Demo + Clinical*	XGBoost	0.51 (0.45–0.57, 0.63)	0.65 (0.59–0.70, <0.05)	0.14 (0.06–0.21, <0.05)	0.74 (0.69–0.79, 0.65)	0.64 (0.59–0.68, 0.59)
Accel*	Squeezenet	0.62 (0.53–0.70, 0.07)	0.75 (0.71–0.79, <0.05)	0.47 (0.35–0.57, <0.01)	0.76 (0.71–0.81, 1.00)	0.72 (0.68–0.76, 0.08)
Accel + Demo**	Resnet	0.62 (0.52–0.69, 1.00)	0.76 (0.71–0.80, 0.76)	0.52 (0.40–0.63, 0.55)	0.74 (0.70–0.78, 0.55)	0.72 (0.68–0.76, 1.00)
Accel + Clinical**	Squeezenet	0.62 (0.52–0.69, 1.00)	0.75 (0.70–0.79, 1.00)	0.49 (0.37–0.57, 0.80)	0.74 (0.70–0.78, 0.55)	0.72 (0.68–0.75, 1.00)
Accel + Demo + Clinical***	Resnet	0.73 (0.63–0.78, 0.06)	0.80 (0.75–0.84, 0.12)	0.60 (0.48–0.70, 0.33)	0.79 (0.74–0.82, 0.08)	0.77 (0.73–0.80, <0.05)

Accel – accelerometer data, demo – demographics (age, sex, race, height, weight, and length of stay), clinical – the clinical set of features (blood pressure, heart rate, spo2, pain score, Braden score, and acute brain dysfunction status). *Indicates that the *p*-values for the setups were calculated by comparison with the SOFA score baseline, **Indicates that the *p*-values for the setups were calculated by comparison with the Accel-only setup, ***Indicates that the *p*-values for the setups were calculated by comparison with the Accel + Clinical setup.

The performance of our baseline SOFA score-based predictor is notably limited, with suboptimal AUC (0.53), precision (0.23), sensitivity (0.30), and F1 score (0.66). However, the model demonstrates a relatively high specificity of 0.76.

Incorporating accelerometer data (Accel) alone or combined with demographic and clinical variables (Accel + Demo, Accel + Clinical, Accel + Demo + Clinical) significantly improved the model’s performance across all metrics. Notably, adding accelerometer data improves AUC, precision, sensitivity, specificity, and F1-score compared to the SOFA score baseline.

Combining accelerometer data with demographic and clinical variables (Accel + Demo + Clinical) yields the best overall performance among the scenarios involving accelerometer data. This model achieves the highest AUC of 0.73, indicating superior discriminative ability compared to other scenarios. Moreover, it exhibits the highest precision (0.80), sensitivity (0.60), specificity (0.79), and F1 score (0.77). Our best setup demonstrated a relatively lower *p*-value.

Optuna provided us with detailed information and hyperparameter selection suggestions. Table 4 outlines the best hyperparameters found by the search for each combination of feature sets. Table A1 comprehensively overviews the hyperparameters and their corresponding values. For the scenario where only accelerometer data was utilized (Accel), SqueezeNet architecture with a batch size of 16, learning rate of 2.11 × 10^−4^, and weight decay of 9.23 × 10^−6^ yielded the best results. The accelerometer downsampling factor was set to 1. Incorporating demographic data along with accelerometer data (Accel + Demo) led to the selection of Resnet architecture with similar hyperparameters, except for a slightly lower learning rate of 1.16 × 10^−4^ and weight decay of 2.77 × 10^−6^. The downsampling factor was adjusted to 2 in this scenario. When clinical data was added to accelerometer data (Accel + Clinical Data), SqueezeNet architecture was again favored, with hyperparameters akin to the Accel scenario, except for a higher weight decay of 9.88 × 10^−4^ Finally, combining accelerometer, demographic, and clinical data (Accel + Demo + Clinical Data) led to the choice of Resnet architecture with a batch size of 16, a learning rate of 9.37 × 10^−5^, and weight decay of 2.13 × 10^−4^. The downsampling factor remained consistent with the Accel + Demo scenario at 2. We achieved the best Accel + Demo + Clinical scenario performance AUC of 0.73 (0.63–0.78).

**Table 4:** Best hyperparameters for each scenario.

	Model	Number of parameters (million)	Batch size	Learning rate	Weight decay	Accelerometer downsampling factor
Accel	Squeezenet	4.33	16	2.11 × 10^−4^	9.23 × 10^−6^	1
Accel + Demo	Resnet	3.90	16	1.16 × 10^−4^	2.77 × 10^−6^	2
Accel + Clinical Data	Squeezenet	5.61	16	2.14 × 10^−4^	9.88 × 10^−4^	1
Accel + Demo + Clinical Data	Resnet	4.21	16	9.37 × 10^−5^	2.13 × 10^−4^	2

**Figure 4:**
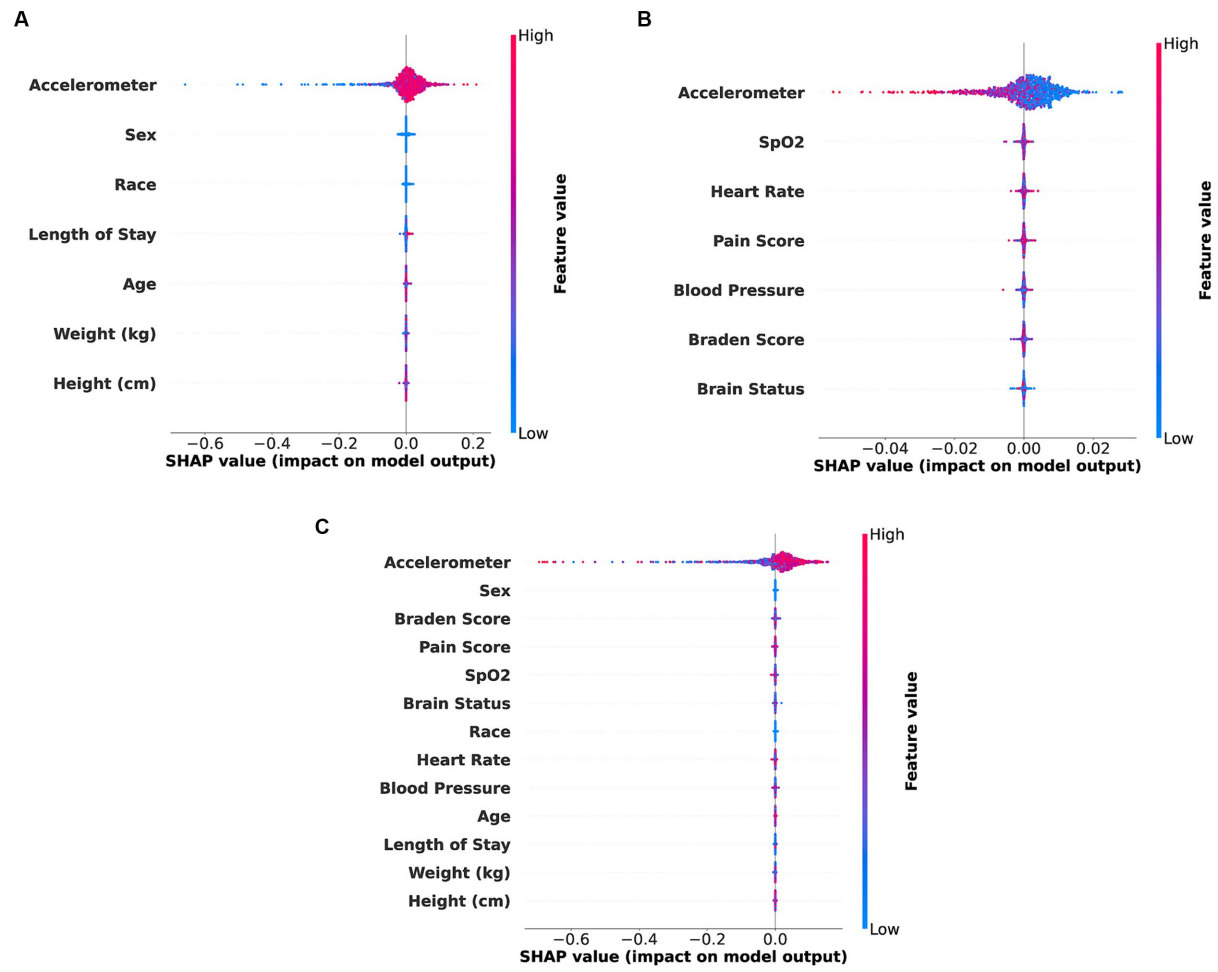
SHAP bee swarm plot illustrating feature importance for different types of feature combinations. **(A)** Accelerometer and demo (Accel + Demo) features. **(B)** Accelerometer and clinical (Accel + Clinical) features. **(C)** Accelerometer, demo and clinical (Accel + Demo + Clinical) features.

Figure 4 illustrates the application of SHAP interpretability analysis in detecting relative feature importance for three specific feature combinations: accelerometer and demographic features, accelerometer and clinical features, and accelerometer clinical and demographic features. This analysis is conducted on the best models obtained for each feature combination scenario. The significance of these features can aid in potential feature selection or assessing their impact on patient diagnosis.

### 3.3 Discussion

This study explored the potential of accelerometry and EHR data in directly determining patients’ acuity state as an alternative to depending exclusively on rule-based scoring systems like the SOFA score. Our analysis revealed that the SOFA score-based predictor exhibited notable limitations, with suboptimal precision, sensitivity, and F1 score, reflecting its inadequacy in effectively evaluating patient conditions. Although the model demonstrated relatively high specificity, its AUC did not significantly surpass random chance, indicating the need for more sophisticated predictive models in clinical practice.

In contrast, incorporating accelerometer data alone or combined with demographic and clinical variables significantly enhanced model performance across all metrics. Notably, adding accelerometer data improved AUC, precision, sensitivity, specificity, and F1 score compared to the SOFA score baseline. These findings underscored the importance of integrating additional features beyond traditional clinical variables for accurate predictive modeling in medical settings. We believe that the additional features encompass aspects of patient physiology and functional status that are not effectively captured by SOFA inputs (or inputs for other traditional models such as APACHE and MEWS). The ability of accelerometer data to capture patient mobility and range of motion continuously can augment the current practice of hourly assessments that are subject to individual bias and is limited to observations of the bedside nurse. Therefore, we are not only enhancing predictive performance but also adding nuance to patient assessment, enriching the overall assessment process. Among the scenarios involving accelerometer data, the model incorporating accelerometer data with demographics and clinical information (Accel + Demo + Clinical) demonstrated the best overall performance. This comprehensive approach yielded the highest AUC, precision, sensitivity, specificity, and F1 score, emphasizing the synergistic benefits of integrating multiple data types for predictive modeling. The robust performance of this model, with highly significant *p*-values, validated its effectiveness in predicting patient outcomes.

All models performed best with maintaining the original frequency of the accelerometer (downsampling scale of 1) which indicated that the long sequence was not a problem for the employed architectures. Notably, a bigger batch size was necessary for the models when clinical data was included in the model. It could indicate that the added complexity introduced by the clinical data required more samples to be processed simultaneously for the model to effectively identify patterns, optimize the gradients, and achieve better convergence during training.

While our study offers valuable insights, it is crucial to acknowledge limitations. Firstly, the generalizability of our findings may be constrained by the size and patient population of mainly white people studied at a single center, warranting validation on diverse datasets to enhance applicability. Despite the clinical research team’s daily checks to ensure proper placement of accelerometer devices and requests to the nursing staff to document the times of device removal and application, it is probable that a small amount of data included in this study’s analyses were recorded while the device was not placed on the patient. The exclusion of patients who died within 24 h of recruitment, coupled with the inability to place study devices on the arms of patients with numerous intravenous and/or intraarterial lines or other equipment (i.e., wrist restraints), may have introduced bias through the exclusion of these high acuity patients from our cohort. Furthermore, the collection of accelerometry data and use of a motion-monitoring system may be unsuited for the acuity assessments of intubated and sedated patients, since the active mobility in these patients is extremely limited.

Finally, it is essential to note that SHAP feature importance is correlated with model performance and may be vulnerable to misclassification due to overfitting, potentially leading to erroneous feature interpretations.

Accelerometer data emerges as an area of high potential for future research endeavors. Its utility extends to evaluating patient mobility, i.e., measuring the ability to change and control body position. Expanding this research to include the integration of additional clinical features, such as medication history, laboratory test results, and admission information, holds potential for further advancements. Moreover, utilizing multimodal models incorporating various pervasive sensing data like depth images, color RGB images, electromyography, sound pressure, and light levels offers opportunities to enhance model performance.

In Figures 4A–C, it is evident that in the combinations of accelerometer with demographic features, accelerometer with clinical features, and all of them together, the accelerometer features exhibit higher importance compared to other features. The accelerometer features demonstrate a broad range of values in positive and negative directions, suggesting its strong indicative nature for acuity analysis, which aligns with our best model results.

Across scenarios utilizing only accelerometer data, accelerometer with demographic data, and accelerometer with clinical data, similar performance was observed on our test data, with an AUC of 0.62 for each combination. It suggests that clinical or demographic features alone, when combined with accelerometer data, do not significantly enhance the models’ ability to classify our dataset. It underscores the critical role of accelerometer data in acuity assessment tasks.

Furthermore, combining accelerometer data with clinical and demographic data improved the AUC from 0.62 to 0.73, indicating an inter-feature dependency among these variables, which benefits our model.

## 4 Conclusion

Critical care environments necessitate the timely assessment of patient acuity to determine the severity of illness and prioritize care accordingly. Our analysis revealed limitations in the SOFA-based predictor, highlighting the need for more sophisticated models in clinical practice. Integrating accelerometer data, either alone or with demographic and clinical variables, significantly enhanced model performance, underscoring the importance of diverse data sources in predictive modeling. The model combining accelerometer data with demographics and clinical information exhibited the highest performance, validating its efficacy in predicting patient acuity. This underscores the importance of a comprehensive approach to patient acuity assessment in critical care settings. While initial findings are promising, further research is imperative to optimize the accuracy and efficiency of these assessments, ensuring advancements in patient care and safety.

It is important to acknowledge that the observational studies for which this data was collected were conducted with the intent of being unobtrusive to patient care, and patients or their proxies were always given the opportunity to opt out of, or discontinue, accelerometer data collection. Additional research is required to ascertain the reliability of mobility data for evaluating intubated and sedated patients. Moreover, further investigation is warranted to evaluate their seamless integration into clinical workflows, ensuring they do not add to nursing workload or physician information overload. Additionally, thoughtful consideration needs to be given to how the outputs and assessments of these models can be communicated effectively, ensuring they offer actionable insights for healthcare providers.

## Data availability statement

The datasets for this article are not publicly available due to concerns regarding participant/patient anonymity. Requests to access the datasets should be directed to the corresponding author.

## Ethics statement

The studies involving humans were approved by University of Florida Institutional Review Board. The studies were conducted in accordance with the local legislation and institutional requirements. The participants provided their written informed consent to participate in this study.

## Author contributions

JS: Data curation, Formal analysis, Investigation, Methodology, Software, Validation, Writing – original draft, Writing – review & editing. MM: Methodology, Software, Visualization, Writing – original draft, Writing – review & editing, Validation. JZ: Software, Writing – original draft, Writing – review & editing. AD: Data curation, Writing – review & editing. SB: Conceptualization, Supervision, Writing – original draft, Writing – review & editing. SN: Writing – review & editing. YR: Writing – review & editing. TO-B: Writing – review & editing. BS: Writing – review & editing. TL: Writing – review & editing. WS: Supervision, Writing – review & editing. AB: Conceptualization, Funding acquisition, Writing – review & editing. PR: Conceptualization, Funding acquisition, Supervision, Writing – review & editing.

## Footnotes

^1^https://theactigraph.com/academic-research#actilife

^2^https://www.consensys.net

## Appendix

Tables A1, A2.

**Table A1:** Overview of the hyperparameters and their respective values explored in the hyperparameter optimization.

Hyperparameter	Values
Model	VGG, ResNet, MobileNet, SqueezeNet, and Transformers
Batch size	8, 16, 24 and 32
Learning rate	Ranging from 10^−5^ to 10^−1^
Weight decay	Ranging from 10^−10^ to 10^−3^
Accelerometer downsampling factor	1, 2, and 4

**Table A2:** Number of parameters and flop counts for each of our models for the accel + demo + clinical data combination with downsampling factor of 1.

Model name	Number of parameters (million)	Flops (G)
VGG	34.6	193.29
ResNet	4.21	158.45
MobileNet	1.06	79.87
SqueezeNet	4.65	173.91
Transformer	0.75	23.01
